# Supplementary material for: DNA Mismatch Repair Gene Variant Classification: Evaluating the Utility of Somatic Mutations and Mismatch Repair Deficient Colonic Crypts and Endometrial Glands
Source: Cancers (Basel). 2023 Oct 10;15(20):4925. doi: 10.3390/cancers15204925 (PMC10605939; doi:10.3390/cancers15204925)
Supplement: Supplementary file 1 [file cancers-15-04925-s001.zip › cancers-2623778-supplementary.pdf]

Supplementary Materials: DNA Mismatch Repair gene Variant Classification: Evaluating the Utility of Somatic Mutations and Mismatch Repair Deficient Colonic Crypts and Endometrial Glands

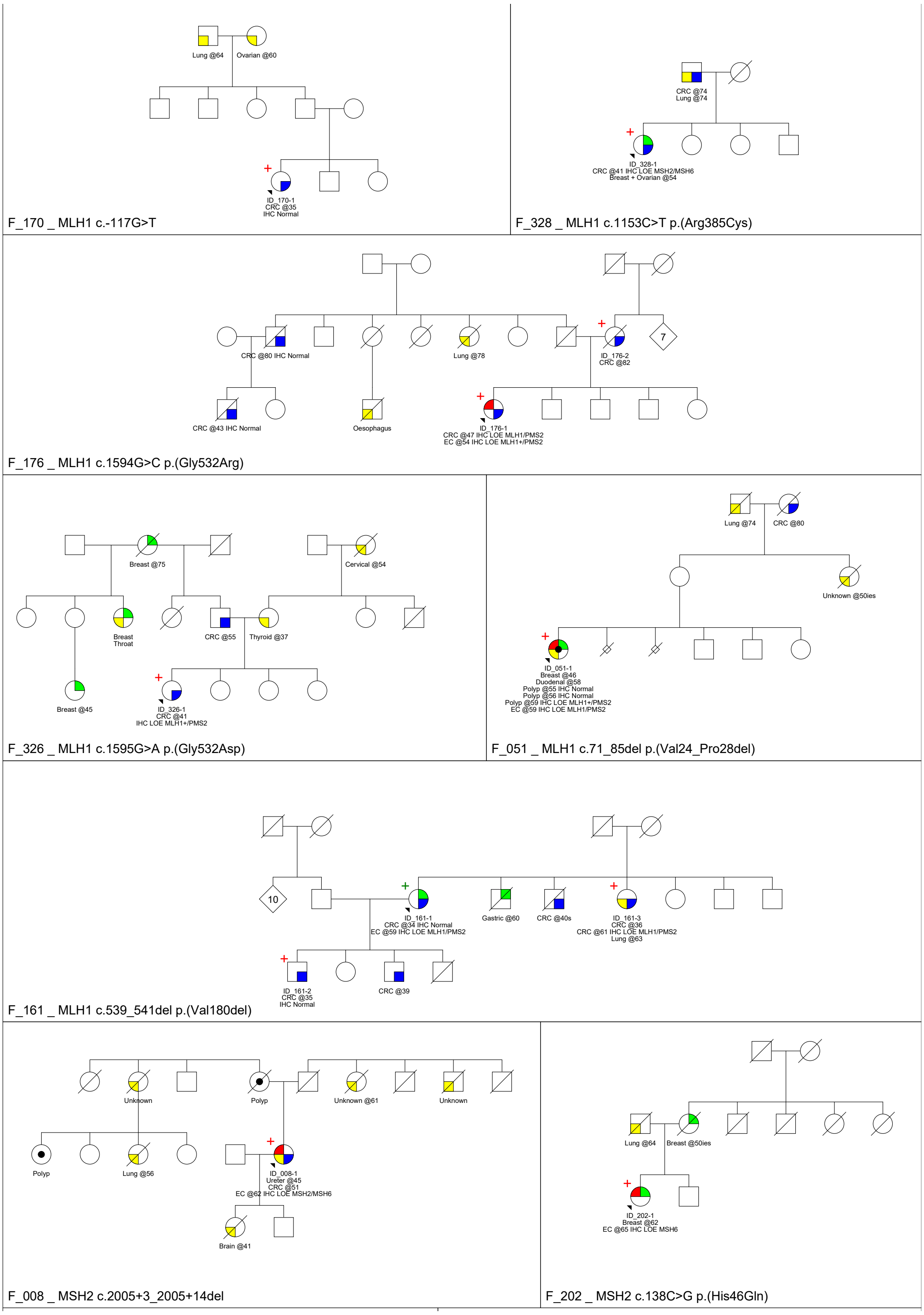

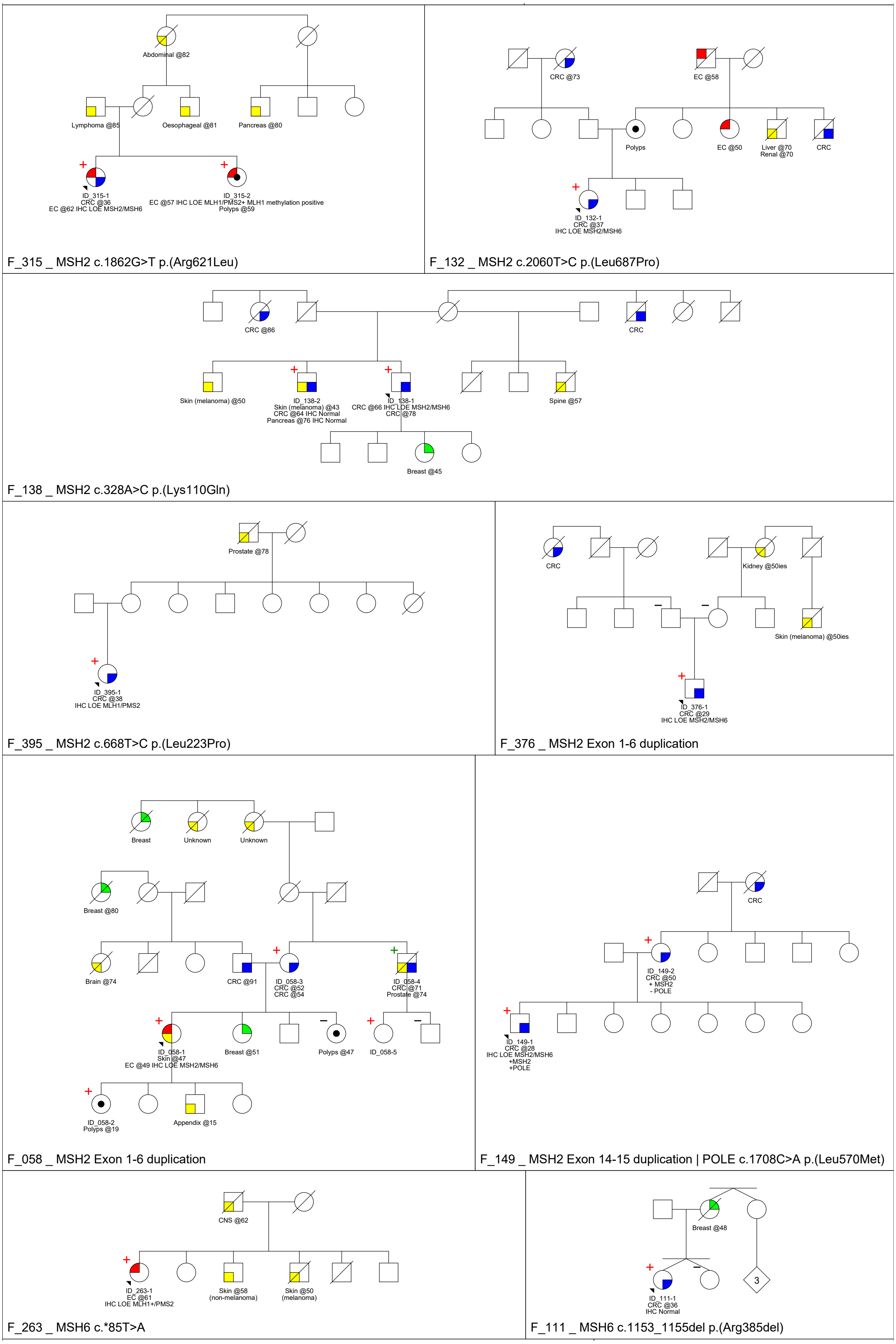
Abdominal @82
Lymphoma @85
Oesophageal @81
Pancreas @80
ID_315-1
CRC @36
EC @62 IHC LOE MSH2/MSH6
ID_315-2
EC @57 IHC LOE MLH1/PMS2+ MLH1 methylation positive
Polyps @59
F_315 _ MSH2 c.1862G>T p.(Arg621Leu)
CRC @73
EC @58
Polyps
EC @50
Liver @70
Renal @70
CRC
ID_132-1
CRC @37
IHC LOE MSH2/MSH6
F_132 _ MSH2 c.2060T>C p.(Leu687Pro)
CRC @86
CRC
Skin (melanoma) @50
ID_138-2
Skin (melanoma) @43
CRC @64 IHC Normal
Pancreas @76 IHC Normal
ID_138-1
CRC @66 IHC LOE MSH2/MSH6
CRC @78
Spine @57
Breast @45
F_138 _ MSH2 c.328A>C p.(Lys110Gln)
Prostate @78
ID_395-1
CRC @38
IHC LOE MLH1/PMS2
F_395 _ MSH2 c.668T>C p.(Leu223Pro)
CRC
Kidney @50ies
Skin (melanoma) @50ies
ID_376-1
CRC @29
IHC LOE MSH2/MSH6
F_376 _ MSH2 Exon 1-6 duplication
Breast
Unknown
Unknown
Breast @80
Brain @74
CRC @91
ID_058-3
CRC @52
CRC @54
ID_058-4
CRC @71
Prostate @74
ID_058-1
Skin @47
EC @49 IHC LOE MSH2/MSH6
Breast @51
Polyps @47
ID_058-5
ID_058-2
Polyps @19
Appendix @15
F_058 _ MSH2 Exon 1-6 duplication
CRC
ID_149-2
CRC @50
+ MSH2
- POLE
ID_149-1
CRC @28
IHC LOE MSH2/MSH6
+MSH2
+POLE
F_149 _ MSH2 Exon 14-15 duplication | POLE c.1708C>A p.(Leu570Met)
CNS @62
ID_263-1
EC @61
IHC LOE MLH1+/PMS2
Skin @58
(non-melanoma)
Skin @50
(melanoma)
F_263 _ MSH6 c.*85T>A
Breast @48
3
ID_111-1
CRC @36
IHC Normal
F_111 _ MSH6 c.1153_1155del p.(Arg385del)

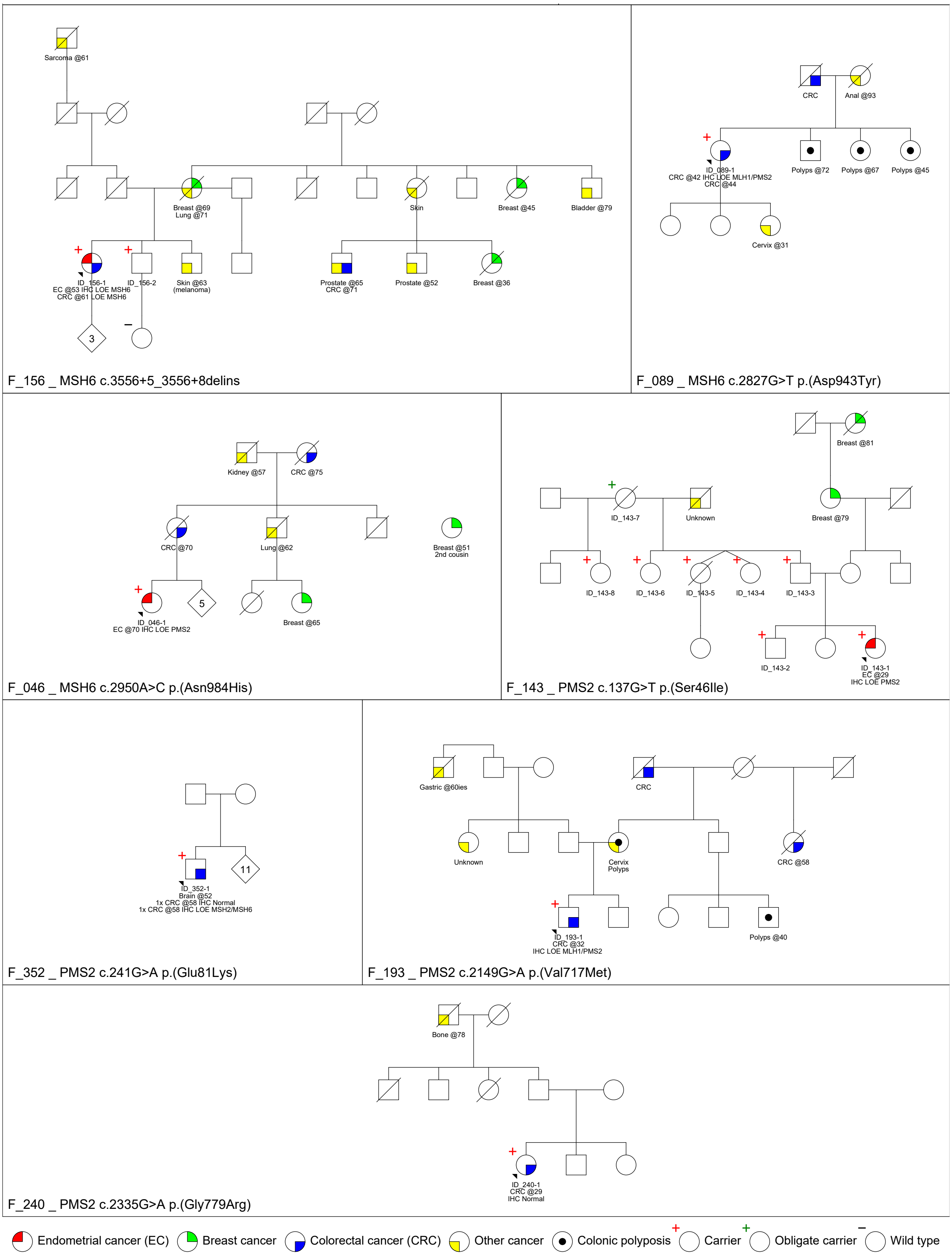

Figure S1. Overview of each of the participants' family pedigree. The explanation for each symbol in the pedigree is outlined within the figure. *Abbreviations*: CRC, colorectal cancer; EC, endometrial cancer.

(A) Reference Group of tumors from Lynch syndrome carriers (n=16) and pMMR non-Lynch tumors (n=18)

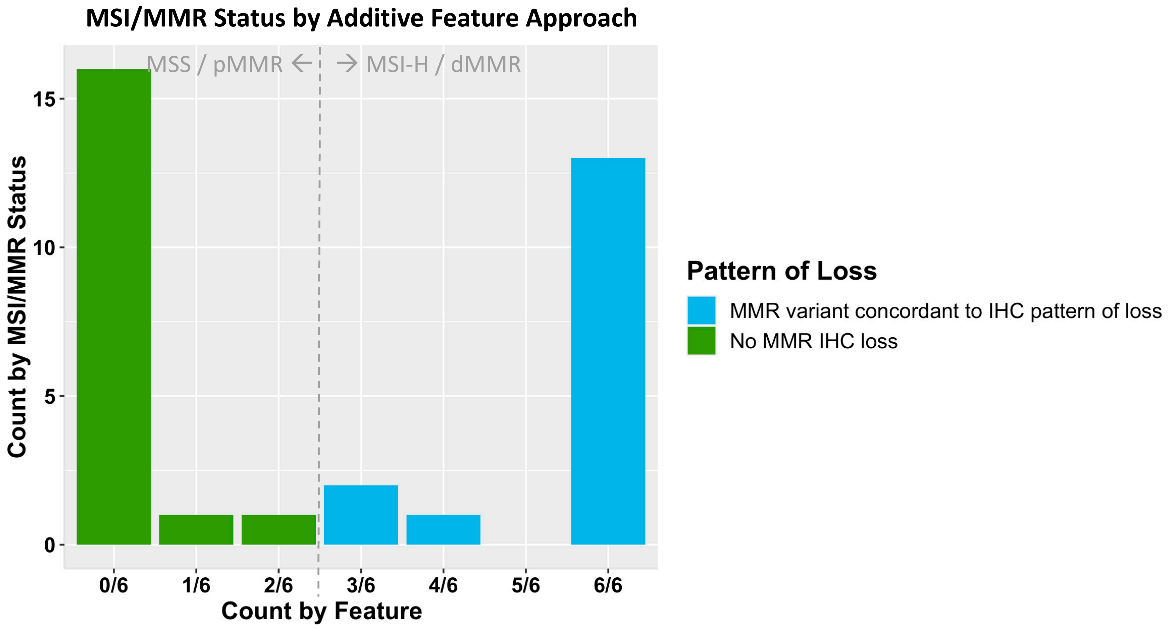

(B) Test Group of n=28 tumors from n=25 VUS carriers tested in this study

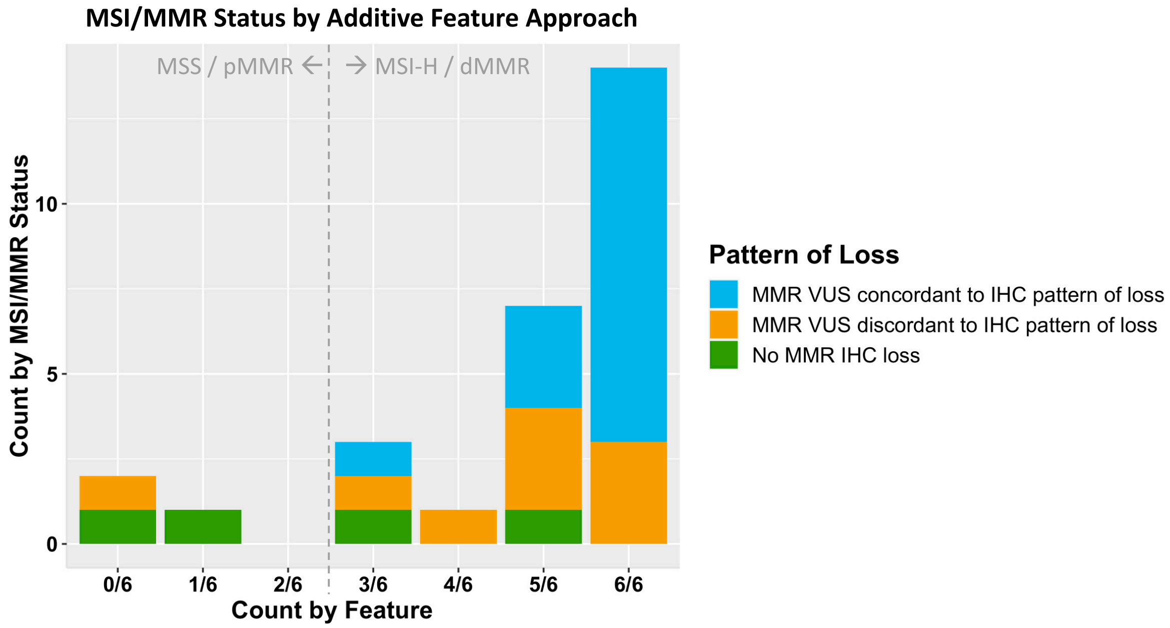

Figure S2. Bar plot presenting the feature count for targeted panel sequenced (A) reference and (B) test groups to determine the DNA mismatch repair status in next-generation sequencing screened tumors after applying the additive feature combination approach. Grey dotted line indicates MSI and MMR status by additive feature combination approach with 0/6, 1/6 and 2/6 indicating MSS/pMMR and 3/6, 4/6, 5/6 and 6/6 indicating MSI-H/dMMR. *Abbreviations:* MMR, DNA mismatch repair; dMMR, DNA mismatch repair deficient; pMMR, DNA mismatch repair proficient; MSI, microsatellite instability; MSI-H, high levels of microsatellite instability; MSS, microsatellite stable; IHC, immunohistochemistry; VUS, variant of uncertain clinical significance.

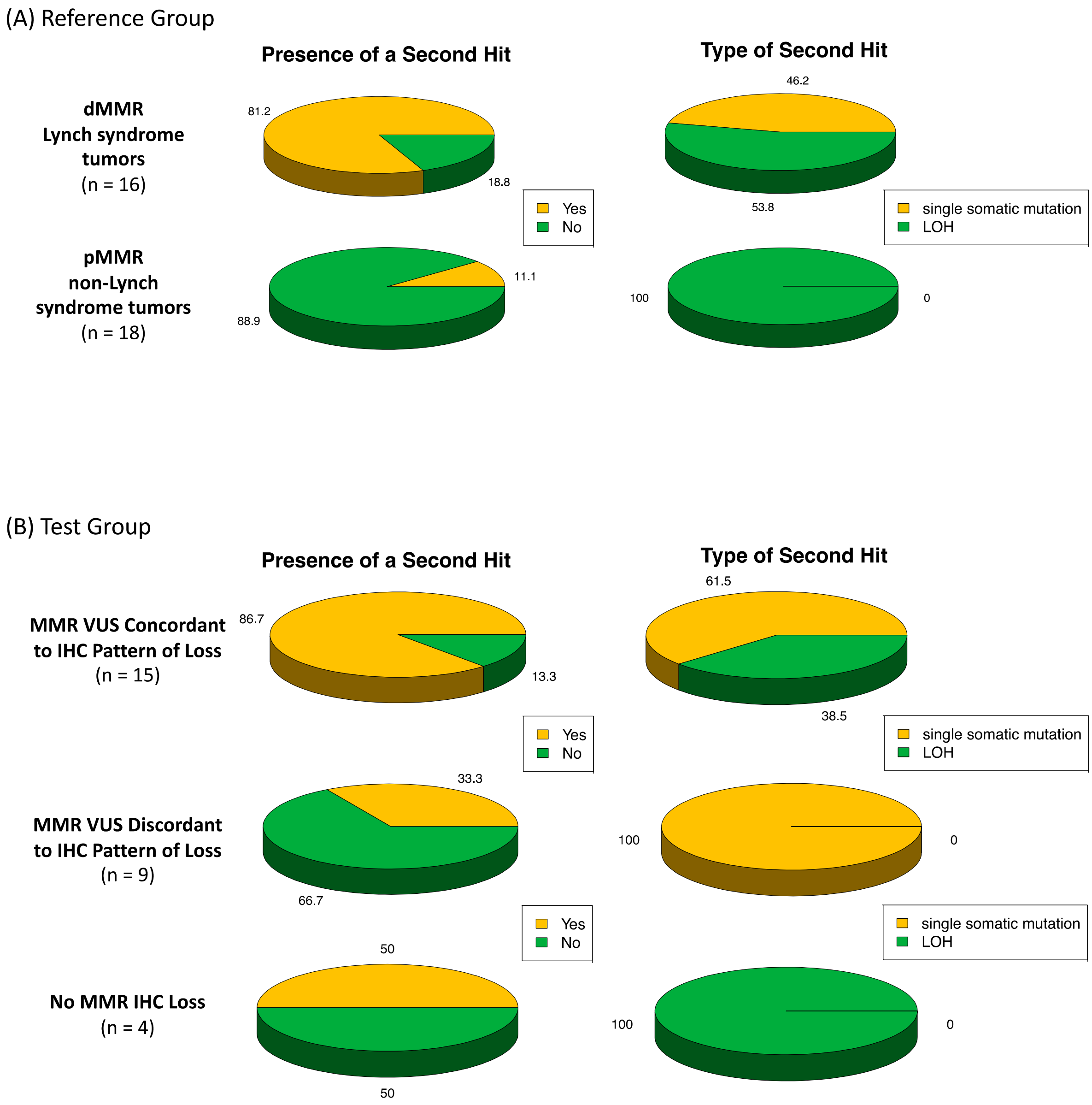

Figure S3. Pie charts presenting the proportions of the presence of a somatic second hit and by which mutation type for (A) the reference and (B) the test groups. Abbreviations: MMR, DNA mismatch repair; dMMR, DNA mismatch repair deficiency, pMMR, DNA mismatch repair proficiency; VUS, variant of uncertain significance; IHC, immunohistochemistry; LOH, loss of heterozygosity.

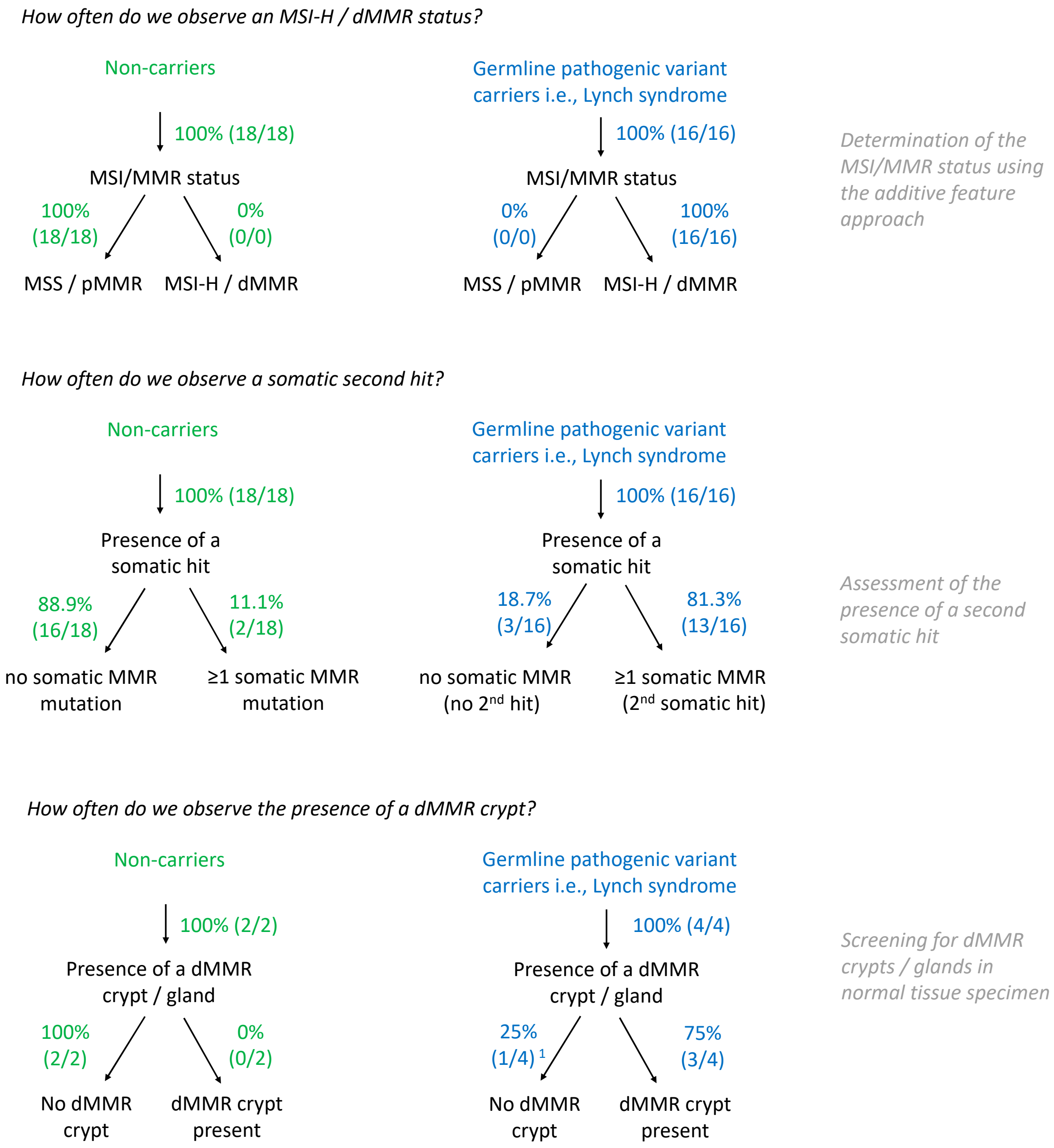

Figure S4. Flowchart displaying the prevalence of the three Lynch syndrome associated features in the reference group. Flowchart incorporating the tumor sequencing and dMMR crypt/gland screening for determining pathogenicity against the ACMG/InSiGHT framework for MMR VUS in the reference group. *Abbreviations*: MMR, DNA mismatch repair; dMMR, DNA mismatch repair deficient; pMMR, DNA mismatch repair proficient; MSI, microsatellite instability; MSI-H, high levels of microsatellite instability; MSS, microsatellite stable; VUS, variant of uncertain clinical significance; NGS, next-generation sequencing; LP, likely pathogenic. [1] Block was depleted after screening of 2x80μM of normal colorectal tissue.

Table S1. Overview of tumors included in the reference group.

Group	#	Carrier ID	Tissue ID	Germline Variant: Gene	Germline Variant: Base Change	Germline Variant: Protein Change	ACMG/ InSiGHT Classification	Tumor Molecular Data: Tissue	Tumor Molecular Data: IHC	Tumor Molecular Data: *MLH1* Methylation	Lynch Syndrome Associated Tissue Features: MSI/MMR Status by Additive Feature Approach	Lynch Syndrome Associated Tissue Features: Presence of a Somatic Second Hit in MMR Gene Harboring VUS	Lynch Syndrome Associated Tissue Features: Presence of a Somatic Mutation NOT in MMR Gene Harboring VUS	Lynch Syndrome Associated Tissue Features: Presence of a dMMR Crypt / Gland	Final Tumour Classification
MMR VARIANT CONCORDANT TO IHC PATTERN OF LOSS	**1**	Ref_109	CRC_109	MLH1	c.116+5G>C	p.?	Class 5: P	CRC	MLH1/PMS2	Negative	MSI-H / dMMR (6/6)	1x LOH (MLH1)	None	NT	dMMR - LS + 2nd hit
	**2**	Ref_811	CRC_811	MLH1	c.350C>T	p.Thr117Met	Class 5: P	CRC	MLH1/PMS2	Negative	MSI-H / dMMR (6/6)	1x LOH (MLH1)	None	NT	dMMR - LS + 2nd hit
	**3**	Ref_412	EC_412	MLH1	c.678G>C	p.?	Class 5: P	EC	MLH1/PMS2	Negative	MSI-H / dMMR (3/6)	1x LOH (MLH1)	1x mut. (MSH6)	NT	dMMR - LS + 2nd hit
	**4**	Ref_400	CRC_400	MLH1	Exon 3 deletion	p.?	Class 5: P	CRC	MLH1/PMS2	Negative	MSI-H / dMMR (6/6)	1x LOH (MLH1)	None	NT	dMMR - LS + 2nd hit
	**5**	Ref_012	CRC_012	MSH2	c.212-478T>G	p.?	Class 4: LP	CRC	MSH2/MSH6	NT	MSI-H / dMMR (4/6)	None	None	NT	dMMR - LS
	**6**	Ref_630	EC_630	MSH2	c.942+3A>T	p.?	Class 4: LP	EC	MSH2/MSH6	Negative	MSI-H / dMMR (6/6)	1x mut. (MSH2)	None	NT	dMMR - LS + 2nd hit
	**7**	Ref_601	EC_601	MSH2	c.1009C>T	p.Gln337*	Class 5: P	EC	MSH2/MSH6	Negative	MSI-H / dMMR (6/6)	1x mut. (MSH2)	1x mut. (MSH6)	NT	dMMR - LS + 2nd hit
	**8**	Ref_500	EC_500	MSH2	c.1705_1706delGA	p.Glu569Ilefs*2	Class 5: P	EC	MSH2/MSH6+	Negative	MSI-H / dMMR (6/6)	1x LOH (MSH2)	None	NT	dMMR - LS + 2nd hit
	**9**	Ref_033	EC_033	MSH2	c.1865C>T	p.Pro622Leu	Class 5: P	EC	MSH2/MSH6	NT	MSI-H / dMMR (6/6)	1x mut. (MSH2)	1x mut. (MSH6)	NT	dMMR - LS + 2nd hit
	**10**	Ref_003	CRC_003	MSH2	c.2005+3_2005+14del	p.?	Class 4: LP	CRC	MSH2/MSH6	NT	MSI-H / dMMR (6/6)	1x LOH (MSH2)	1x mut. (MLH1) and 1x mut. (PMS2)	NT	dMMR - LS + 2nd hit
	**11**	Ref_036	EC_036	MSH2	c.2502_2508del	p.Asn835Leufs*4	Class 5: P	EC	MSH2/MSH6	Negative	MSI-H / dMMR (6/6)	None	None	NT	dMMR - LS
	**12**	Ref_404	CRC_404	MSH2	Exon 1-6 deletion	p.?	Class 5: P	CRC	MSH2/MSH6	NT	MSI-H / dMMR (3/6)	None	1x mut. (MSH6) and 1x mut. (PMS2)	NT	dMMR - LS
	**13**	Ref_600	CRC_600	MSH2	Exon 90 deletion	p.?	Class 5: P	CRC	MSH2/MSH6	NT	MSI-H / dMMR (6/6)	1x mut. (MSH2)	1x mut. (MLH1)	NT	dMMR - LS + 2nd hit
	**14**	Ref_704	CRC_704	MSH2	10 megabase inversion	p.?	Class 5: P	CRC	MSH2/MSH6	NT	MSI-H / dMMR (6/6)	1x mut. (MSH2)	2x mut. (MSH6)	NT	dMMR - LS + 2nd hit
	**15**	Ref_010	CRC_010	MSH6	c.3311_3312del	p.Phe1104Trpfs*3	Class 5: P	CRC	MSH6	NT	MSI-H / dMMR (6/6)	1x LOH (MSH6)	None	NT	dMMR - LS + 2nd hit
	**16**	Ref_631	CRC_631	PMS2	Exon 6-8 deletion	p.?	Class 5: P	CRC	PMS2	NT	MSI-H / dMMR (6/6)	1x mut. (PMS2)	None	NT	dMMR - LS + 2nd hit
	**17**	Ref_029[1]	CRC_029	MLH1	c.1713_1716del	p.Phe571Leufs*19	Class 5: P	CRC	MLH1/PMS2	Negative	MSI-H / dMMR (5/6)	1x mut. (MLH1)	1x mut. (MSH6)	Yes	dMMR - LS + 2nd hit
	**18**	Ref_605[1]	CRC_605	MLH1	c.1852_1854del	p.Lys618del	Class 5: P	CRC	MLH1/PMS2	Negative	MSI-H / dMMR (6/6)	1x mut. (MLH1)	None	Yes	dMMR - LS + 2nd hit
	**19**	Ref_411[1]	CRC_411	MSH2	c.1889_1892del	p.Gly630Glufs*4	Class 5: P	CRC	MSH2/MSH6	NT	MSI-H / dMMR (6/6)	1x mut. (MSH2)	2x mut. (MSH6)	No[2]	dMMR - LS + 2nd hit
NO MMR IHC LOSS	**20**	Ref_061	CRC_061	-	Wildtype	Wildtype	NA	CRC	Normal	NT	MSS / pMMR (1/6)	NA	1x LOH (MLH1)	NT	pMMR
	**21**	Ref_237	CRC_237	-	Wildtype	Wildtype	NA	CRC	Normal	NT	MSS / pMMR (0/6)	NA	1x LOH (MSH6)	NT	pMMR
	**22**	Ref_039	CRC_039	-	Wildtype	Wildtype	NA	CRC	Normal	Negative	MSS / pMMR (2/6)	NA	None	NT	pMMR
	**23**	Ref_092	CRC_092	-	Wildtype	Wildtype	NA	CRC	Normal	Negative	MSS / pMMR (2/6)	NA	None	NT	pMMR
	**24**	Ref_363	EC_363	-	Wildtype	Wildtype	NA	EC	Normal	Negative	MSS / pMMR (0/6)	NA	None	NT	pMMR
	**25**	Ref_082	EC_082	-	Wildtype	Wildtype	NA	EC	Normal	Negative	MSS / pMMR (0/6)	NA	None	NT	pMMR
	**26**	Ref_016	CRC_016	-	Wildtype	Wildtype	NA	CRC	Normal	NT	MSS / pMMR (2/6)	NA	None	NT	pMMR
	**27**	Ref_049	CRC_049	-	Wildtype	Wildtype	NA	CRC	Normal	NT	MSS / pMMR (1/6)	NA	None	NT	pMMR
	**28**	Ref_211	CRC_211	-	Wildtype	Wildtype	NA	CRC	Normal	NT	MSS / pMMR (0/6)	NA	None	NT	pMMR
	**29**	Ref_147	CRC_147	-	Wildtype	Wildtype	NA	CRC	Normal	NT	MSS / pMMR (0/6)	NA	None	NT	pMMR
	**30**	Ref_139	CRC_139	-	Wildtype	Wildtype	NA	CRC	Normal	NT	MSS / pMMR (2/6)	NA	None	NT	pMMR
	**31**	Ref_259	CRC_259	-	Wildtype	Wildtype	NA	CRC	Normal	NT	MSS / pMMR (0/6)	NA	None	NT	pMMR
	**32**	Ref_252	CRC_252	-	Wildtype	Wildtype	NA	CRC	Normal	NT	MSS / pMMR (0/6)	NA	None	NT	pMMR
	**33**	Ref_241	CRC_241	-	Wildtype	Wildtype	NA	CRC	Normal	NT	MSS / pMMR (2/6)	NA	None	NT	pMMR
	**34**	Ref_107	EC_107	-	Wildtype	Wildtype	NA	EC	Normal	NT	MSS / pMMR (0/6)	NA	None	NT	pMMR
	**35**	Ref_055	EC_055	-	Wildtype	Wildtype	NA	EC	Normal	NT	MSS / pMMR (0/6)	NA	None	NT	pMMR
	**36**	Ref_284	EC_284	-	Wildtype	Wildtype	NA	EC	Normal	NT	MSS / pMMR (0/6)	NA	None	NT	pMMR
	**37**	Ref_402	EC_402	-	Wildtype	Wildtype	NA	EC	Normal	NT	MSS / pMMR (0/6)	NA	None	NT	pMMR
	**38**	Ref_897[3]	CRC_897	-	Wildtype	Wildtype	NA	CRC	Normal	NT	NT	NT	NT	No	pMMR
	**39**	Ref_972[3]	CRC_972	-	Wildtype	Wildtype	NA	CRC	Normal	NT	NT	NT	NT	No	pMMR

Abbreviations: ID, identification number; CRC, colorectal cancer; EC, endometrial cancer; IHC, immunohistochemistry; P, pathogenic; LP, likely pathogenic; MMR, DNA mismatch repair; MSI-H, high levels of microsatellite stability; MSS, microsatellite stable; dMMR, DNA mismatch repair deficient; pMMR, DNA mismatch repair proficient; LOH, loss of heterozygosity; mut., single somatic mutation; NA, not applicable; NT, not tested; LS, Lynch syndrome. + Indicates heterogeneous / patchy loss of DNA mismatch repair protein expression by IHC; 1 These samples have undergone whole-exome sequencing; 2 Block was depleted after screening of 2x80µM of normal tissue; 3 These samples did not undergo next-generation sequencing.
